# Impact of Tuberculosis on People Living With HIV: A Deadly Enemy Revealed in a Cross-Sectional Study of the Colombian Caribbean

**DOI:** 10.7759/cureus.93515

**Published:** 2025-09-29

**Authors:** Luis Fernando Saldarriaga Osuna, Felipe A. Muñoz Rossi, Jean Pierre Matias Acosta, Luis Felipe Franco Puente, Natalia Marino Santos, Antonio M Zumaque Carrascal, Edwin Zuniga Simancas

**Affiliations:** 1 General Medicine, National University of Colombia, Bogotá, COL; 2 Internal Medicine, National University of Colombia, Bogotá, COL; 3 Intensive Care Unit, Clinica General del Norte, Barranquilla, COL; 4 General Medicine, Universidad del Sinú, Montería, COL; 5 Internal Medicine, Universidad Pedagògica Y Tecnològica de Colombia, Tunja, COL; 6 General Medicine, Universidad del Sinú, Monteria, COL; 7 Internal Medicine, Universidad del Sinú, Montería, COL; 8 Internal Medicine, Cardiovascular and Metabolic Center, Arjona, COL

**Keywords:** co-infection, hiv, mortality, risk factors, tuberculosis

## Abstract

Background: The human immunodeficiency virus (HIV) destroys CD4 lymphocytes and weakens the immune system, exposing the body to opportunistic infections. People with impaired cellular immunity due to HIV are at greater risk of complications from tuberculosis (TB), which implies a poor health prognosis. When diagnostic and therapeutic interventions are not performed promptly, the outcome can be fatal.

Objective: This study aimed to analyze the epidemiological behavior and factors associated with mortality in TB/HIV coinfection during the years 2015 to 2024 in the Department of Córdoba (Colombia).

Methodology: A cross-sectional analytical study was conducted using retrospective data obtained from event 813 notification forms from the National Public Health Surveillance System (Sivigila). Clinical and sociodemographic data, as well as laboratory reports, were collected. The information is described using graphical methods and numerical indices (measures of central tendency and dispersion). Categorized qualitative and quantitative variables were analyzed using contingency tables, chi-square tests, and multiple logistic regression analysis.

Results: The sociodemographic variables studied were not statistically significant. The variables “hospitalization,” “type of case confirmation,” and “smear microscopy result” were found to have a statistically significant association (p < 0.05) with the final event of interest (deceased or alive).

Conclusions: An explanatory statistical model for mortality in patients with HIV-TB coinfection was evaluated for the first time in the Department of Córdoba (Colombia). Hospitalization and clinical confirmation of the case are associated with a higher risk of mortality. Further research is needed, utilizing different design techniques and fewer limitations, to corroborate the proposed findings.

## Introduction

Tuberculosis (TB) is a bacterial infection caused by *Mycobacterium tuberculosis*, which predominantly affects the pulmonary system, although it can compromise other organs. Human immunodeficiency virus (HIV) infection progressively deteriorates the immune system by destroying CD4 lymphocytes, key cells in the immune response. Coinfection with TB and HIV has a bidirectional impact: HIV reduces the CD4 lymphocyte population, which favors the progression and clinical severity of TB, while TB accelerates the progression of HIV to acquired immunodeficiency syndrome (AIDS), increasing associated mortality [1].

According to the guidelines of the Colombian Ministry of Health for the national TB prevention and control program, TB cases are defined as confirmed by bacteriology or clinical findings. The first is a case that is confirmed by a positive result in one of the laboratory tests, such as smear microscopy, liquid culture, or molecular testing. The second is a case that, despite negative bacteriological tests, is diagnosed based on suggestive clinical symptoms accompanied by abnormalities in radiographic examinations (plain radiography or tomography), suggestive histopathology, or epidemiological link (contact with a confirmed case of TB) [2].

Since the beginning of the HIV epidemic, 88.4 million people have been infected (95% CI 71.3-112.8), and 42.3 million (95% CI 35.7-51.1) have died from HIV-related illnesses. An estimated 39.0 million (95% CI 33.1-45.7) people were living with the virus worldwide in 2022, of whom 1.3 million acquired it in 2023 and 630,000 died from AIDS-related illnesses [3]. In 2023, 10.8 million cases of TB were reported worldwide (95% CI: 10.1-11.7 million), with five countries accounting for 56% of the global total (India 26%, Indonesia 10%, China 6.8%, Philippines 6.8%, and Pakistan 6.3%). TB caused 1.25 million deaths (95% CI: 1.13-1.37 million) in 2023, including 1.09 million among HIV-negative people and 161,000 among people with HIV [4].

In 2024, 185,904 people were reported to be living with HIV in Colombia, of whom 14,555 were new cases during that year. Among the AIDS-defining illnesses, the most common were HIV-associated wasting syndrome (12.91%), pulmonary or extrapulmonary TB (7.20%), and cerebral toxoplasmosis (5.02%). The territorial entities with the highest number of HIV and TB/HIV co-infection cases were Bogotá, Antioquia, and Valle del Cauca. There were 15,310 cases of active TB documented in Colombia in 2024, which corresponds to an increase of 13.71% compared to the previous period (2023), of which 13.28% were coinfected with HIV. In terms of TB/HIV co-infection cases, in 2024, the Department of Córdoba ranked third in the Colombian Caribbean region and seventh nationally [5].

Some research related to HIV has been identified in the Department of Córdoba. Noteworthy is the study that identifies the prevalence of syphilis in HIV patients [6], the study that characterizes the quality of life and support network of HIV-positive patients [7], and another that compares the prevalence of HIV-positive pregnancies in urban and rural areas [8]. Some studies have also been conducted that characterize the behavior of TB in the department [9,10]. However, no research has been conducted to evaluate the behavior of TB/HIV coinfection in the Department of Córdoba, despite its high number of coinfection cases compared to other territorial entities. Studying this comorbidity for the first time in this area of the country will inform the design of strategies to minimize the transmission chain, reduce the disease burden, and improve health outcomes for the department's population.

## Materials and methods

We conducted an observational, analytical, cross-sectional study with retrospective data collection to identify risk factors associated with mortality in patients with TB/HIV coinfection in the Department of Córdoba, Colombia, between 2015 and 2024.

Data were obtained from notification forms corresponding to event 813 of the national epidemiological surveillance system (Sivigila), maintained by the Tuberculosis Control Program of the Córdoba Department Health Secretariat. All reported cases of TB/HIV coinfection during the study period were included without exclusions.

Data were extracted from Sivigila forms, processed in Microsoft Excel (Microsoft Corp., USA), and analyzed using R software version 4.5.1. Variables were primarily categorical, except for age, which was analyzed both as a continuous variable and stratified into three groups: youth (18-28 years), adults (29-59 years), and elderly (≥60 years).

Patients were categorized into two groups according to outcome (deceased vs. non-deceased). Descriptive statistics were generated using frequency tables. Bivariate analysis was performed using chi-square tests.

For multivariate analysis, multiple logistic regression was used. An initial full model with all covariates was refined through backward elimination using the Akaike Information Criterion (AIC). Model performance was assessed with receiver operating characteristic (ROC) curves and validated with accuracy, precision, sensitivity, specificity, and F1-score metrics. Associations were expressed as odds ratios (ORs) and prevalence ratios (PRs) with corresponding confidence intervals.

Ethical considerations

This study is a risk-free investigation, in accordance with the ethical principles of the Helsinki report. The protection of patients' rights and well-being is ensured by articles 11 and 05 of Resolution 8430 of 1993 of the Republic of Colombia. As this was an observational study using secondary data sources, no informed consent documents were completed by the patients. Authorization was obtained from the Tuberculosis Control Program of the Health Secretariat of the Department of Córdoba. The identity of the patients was not disclosed, and their right to privacy was protected by Statutory Law 1581 of 2012 of the Republic of Colombia.

## Results

Sociodemographic characteristics and diagnostic aspects of TB/HIV coinfection

**Figure 1:**
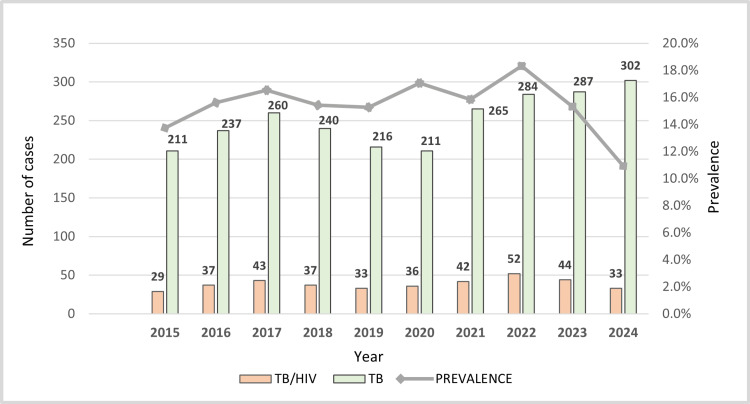
Distribution of number of cases per year of study in patients co-infected with TB/HIV from 2015 to 2024 in Córdoba, Colombia (n = 386).

The patterns of TB and TB/HIV coinfection showed similar fluctuations during the study period (Figure 1). Between 2015 and 2024, the Tuberculosis Control Program of the Córdoba Department Health Secretariat reported 2,513 cases diagnosed with TB and 386 cases diagnosed with TB/HIV coinfection. An overall prevalence of coinfection of 15.36% was calculated during the study period. The average age of patients was found to be 38.31 years (SD 13.78) with a median of 35 years, with middle-aged adults (age group 29-59 years) being the most affected by TB/HIV co-infection (n= 263, 67.96%) and males being the most affected (male-female ratio 3-1).

Table 1 describes the demographic characteristics, comorbidities, and laboratory test results for the study population.

**Table 1:** Sociodemographic and clinical characteristics of patients with TB/HIV coinfection (n = 386).

Variable	n (%)
Age	
0–17 years	7 (1.81)
18–59 years	353 (91.45)
60 or older	26 (6.74)
Sex	
Female	91 (23.58)
Male	295 (76.42)
Area of occurrence	
Urban	309 (80.05)
Rural	77 (19.95)
Hospitalization	
Yes	291 (75.39)
No	95 (24.61)
Type of TB	
Pulmonary	300 (77.72)
Extrapulmonary	86 (22.28)
Type of extrapulmonary TB	
Pleural	19 (4.92)
Meningeal	16 (4.15)
Peritoneal	1 (0.26)
Lymphatic	39 (10.10)
Renal	1 (0.26)
Intestinal	1 (0.26)
Other	9 (2.33)
Not applicable	300 (77.72)
Health insurance scheme	
Subsidized	287 (74.35)
Contributory	69 (17.88)
Special	9 (2.33)
Exception	7 (1.81)
Uninsured	14 (3.63)
Treatment history	
New	342 (88.60)
Previously treated	44 (11.40)
Special population	
Indigenous	8 (2.07)
Migrant	14 (3.63)
Incarcerated	21 (5.44)
Smear microscopy	
Positive	100 (26.68)
Negative	156 (40.41)
Not performed	130 (32.90)
Culture	
Positive	34 (8.81)
Negative	48 (12.44)
In process	43 (11.14)
Not performed	261 (67.62)
Molecular test	
Positive	47 (12.18)
Negative	38 (9.84)
Not performed	301 (77.98)
Histopathology	
Positive	24 (6.22)
Negative	3 (0.78)
Not performed	359 (93.01)
Case confirmation	
By laboratory	182 (47.15)
By clinical criteria	204 (52.85)
Diabetes	
Yes	8 (2.07)
No	309 (80.05)
Unknown	69 (17.88)
Renal disease	
Yes	15 (3.89)
No	302 (78.24)
Unknown	69 (17.88)
Malnutrition	
Yes	49 (12.69)
No	219 (56.74)
Unknown	118 (30.57)

Regarding the diagnosis of the disease, there was a clear predominance of clinically confirmed TB cases (n = 204, 52.85%) compared to microbiologically confirmed cases (n = 182, 47.15%). According to the laboratory tests used, smear microscopy was performed in 256 (67.09%) of cases, with positive results in 100 (39.06%) of tests; culture was performed in 125 (32.39%) of cases, with positive results in 34 (27.2%) of the studies; and molecular testing was performed in 85 (22.02%) of cases, with positive results in 47 (55.29%) of the tests. Histopathology confirmed the diagnosis of TB in 24 (6.21%) of cases. The species Mycobacterium tuberculosis and Mycobacterium bovis were identified in 60 (15.54%) and 4 (1.04%) of confirmed TB cases, respectively.

Analysis of mortality in TB/HIV coinfection

**Figure 2:**
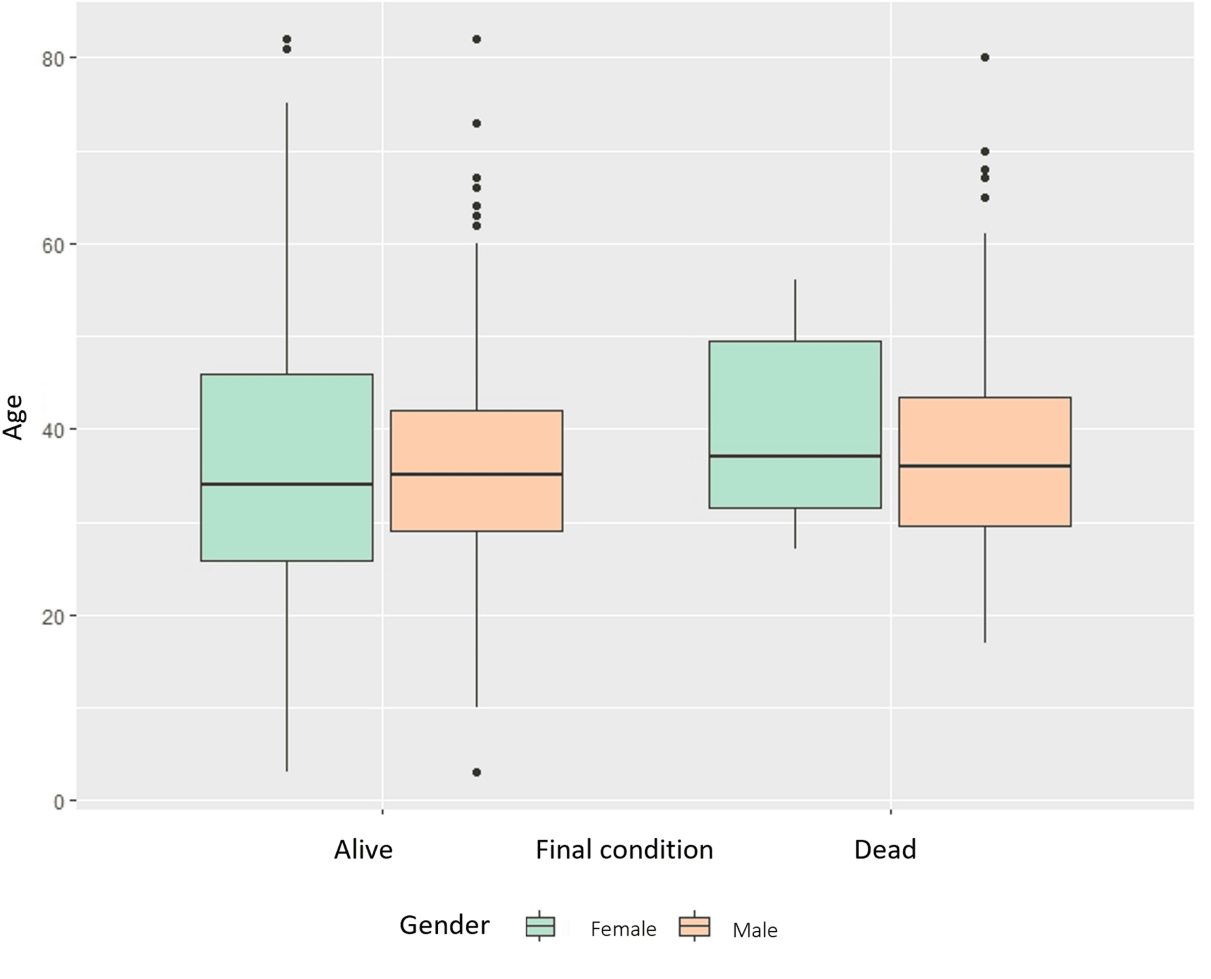
Box and whisker plot. Mortality and age grouped by sex in patients co-infected with TB/HIV from 2015 to 2024 in Córdoba, Colombia (n = 386).

During the observation period, 98 (25.39%) patients died. More men (75 (75.53%)) than women (23 (23.47%)) were affected. A box-and-whisker plot (Figure 2) was used to assess differences in age by sex and outcome (death or survival).

The median age of the women who died was 36.0 years, with a range of 27 to 56 years. The mean age was 38.48 years (95% CI: 24.64-52.32). The average age of women who lived, on the other hand, was 38.2 years (95% CI: 24.44-52.0). The deceased men were between 17 and 80 years old, with a median age of 35.5 years and a mean age of 38.32 years (95% CI: 24.51-52.13). The average age of the men who lived was also 38.2 years (95% CI: 24.56-52.36). The differences between the groups were not statistically significant, but these observations suggest that there may be a link between age and death (age: χ² = 0.49, p = 0.783; sex: χ² = 1.441e−29, p = 1.000).

We used a chi-square test to assess if there were any links between each independent variable and the outcome variable, which we called the "final condition." A p-value threshold of less than 0.05 was considered statistically significant at a 95% confidence level. Table 2 shows that the variable "hospitalization" had the strongest link to the outcome (p = 1.431e−10), followed by "type of case confirmation" (p = 0.0002334).

**Table 2:** Bivariate analysis in patients co-infected with TB/HIV from 2015 to 2024 in Córdoba, Colombia (n = 386).

Variable	Chi-squared (X²)	df	P-value
Age	0.49	2	0.7827
Sex	1.4412 × 10⁻²⁹	1	1.0000
Area of residence	1.3368	1	0.2476
Health insurance scheme	5.7178	4	0.2212
Migrant status	0.4350	1	0.5095
Incarceration	0.1839	1	0.6681
Indigenous ethnicity	0.1482	1	0.7003
Diabetes mellitus	0.6435	2	0.7249
Chronic kidney disease	0.5283	2	0.7679
Malnutrition	3.8142	2	0.1485
Case confirmation method	13.541	1	0.0002
Hospitalization	41.120	1	1.431 × 10⁻¹⁰
Type of TB	0.1406	1	0.7077
Type of extrapulmonary TB	12.527	7	0.0845
History of previous treatment	1.1299 × 10⁻³⁰	1	1.0000
Smear test result	5.3681	2	0.0683
Culture result	1.4033	3	0.7048
Molecular test result	5.7522	2	0.0564
Histopathology result	5.0528	2	0.0800

Then, a multiple logistic regression analysis was done to find factors that predict death. First, a generalized model that included all the independent variables was made. By successively eliminating non-significant variables, several reduced models were produced using the backward elimination technique. The best reduced model, which was chosen based on the lowest AIC, had an AIC value of 364.76. It kept the covariates of hospitalization, histopathology result, smear microscopy result, and type of case confirmation.

**Figure 3:**
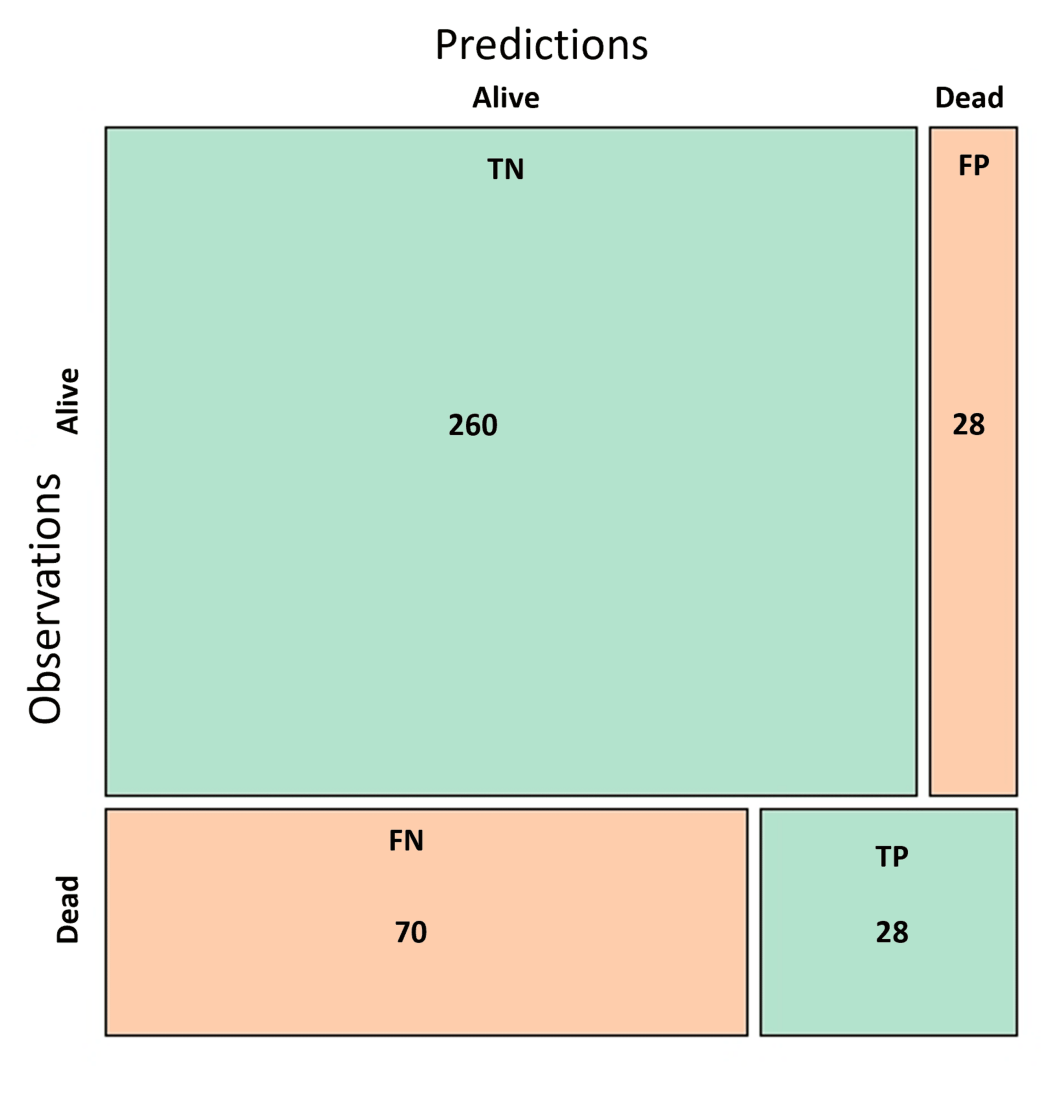
Confusion matrix of the reduced explanatory model. Multivariate analysis in patients coinfected with HIV-TB from 2015 to 2024 in Córdoba, Colombia (n = 386).

Using this last model, a contingency table was built comparing the expected and actual outcomes (dead or alive) (Figure 3). An overall accuracy of 74.61%, a precision of 50%, a specificity of 90.28%, a sensitivity (recall) of 28.57%, and an F1 score of 36.36% were obtained.

Prevalence ratios (PRs) were estimated using the exponential function of the coefficients obtained from the final reduced model. This measure was selected due to the cross-sectional nature of the study, which did not include longitudinal follow-up. As shown in Table 3, the variable type of case confirmation exhibited the strongest association with mortality (p = 0.033), followed by smear test result (p = 0.041). 

**Table 3:** Model fit measures: zero deviation = 437.39, residual deviation = 350.76, AIC = 364.76, R2Cox = 0.201.

Variables	Coefficients	PR	95% CI	P-value
Type of case confirmation	-0.667	0.513	0.274–0.941	0.033
Hospitalization	17.896	5.916 × 10⁷	8.746 × 10²⁷–1.818 × 10¹¹⁹	0.978
Smear test result	0.601	1.824	1.026–3.265	0.041
Histopathology result	17.869	5.765	3.726 × 10⁻²⁰⁴–2.893 × 10⁻¹⁰⁷	0.996

**Figure 4:**
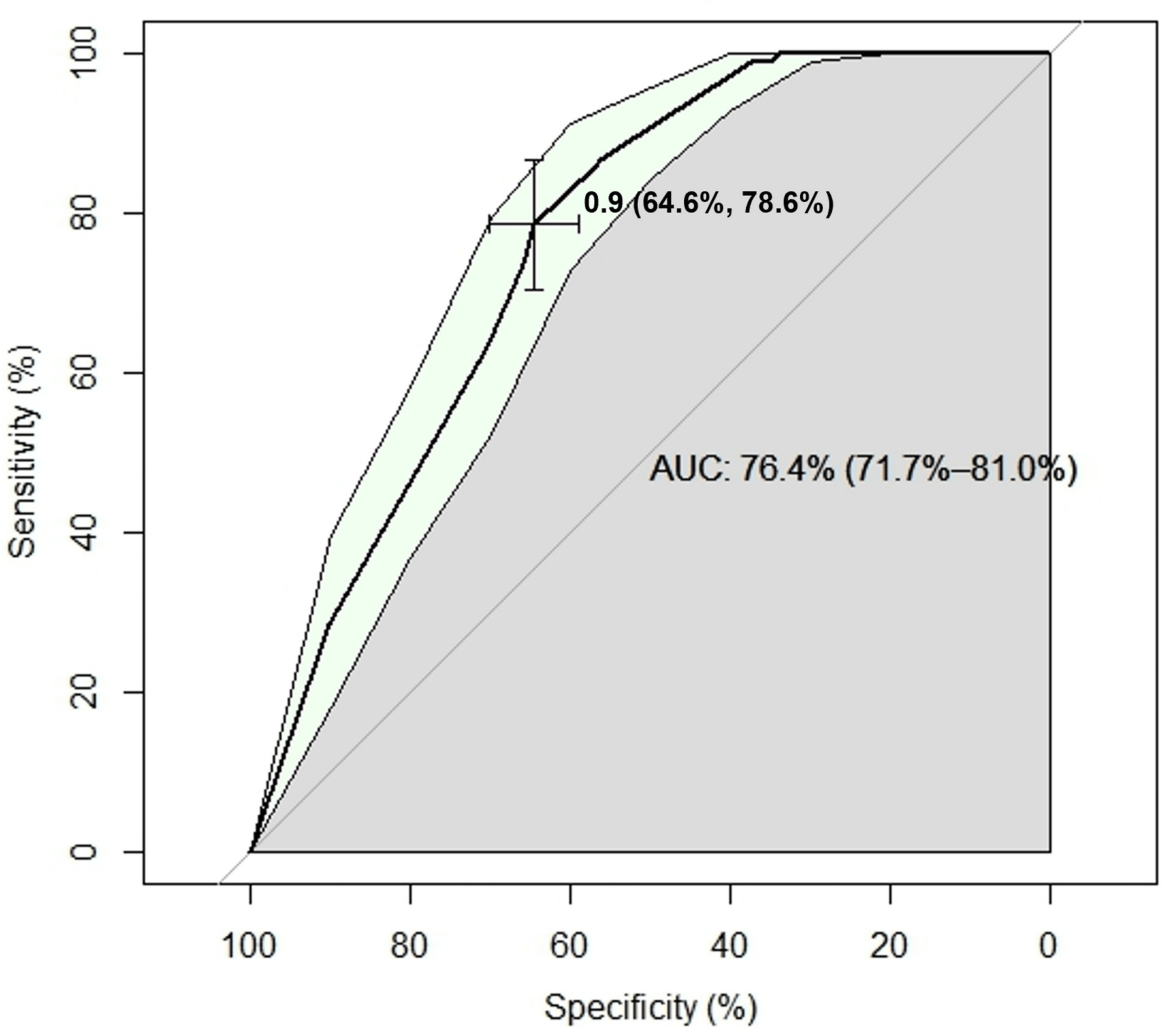
ROC curve of the reduced explanatory model. Patients coinfected with HIV-TB from 2015 to 2024 in Córdoba, Colombia (n = 386).

A receiver operating characteristic (ROC) curve was calculated to evaluate the model's discriminative power (Figure 4), and the results showed an area under the curve (AUC) of 76.4% (95% CI: 71.7-81.0%). The best cutoff point was 0.9, with a sensitivity of 64.6% and a specificity of 78.6%.

## Discussion

Our research reveals a high prevalence of TB/HIV coinfection (368; 15.36%) in the Department of Córdoba during the data collection period, even higher than the national figures reported by the surveillance system in 2022 (2,219; 12.7%) and by the high-cost account in 2024 (2,033; 13.28%) [5,11]. In 2022, a higher incidence of TB cases and TB/HIV coinfection was observed, followed by a subsequent downward trend in cases for both conditions. Probably due to the COVID-19 pandemic, in 2020, there were fewer TB cases than in the other regions of the country.

In this study, it is evident that TB/HIV coinfection primarily affects young adults, with a mean age of 38.31 years (95% CI 24.63-52.09) and a median age of 35 years; the 29-59 age group is the most affected (263; 67.96%), with the predominance of coinfection in men (295; 76.42%). These findings are similar to those reported in different regional and international studies [6-7] and may be related to behavioral and lifestyle differences between the two genders [8].

Due to the retrospective nature of the study, it was not possible to determine the case fatality rate; however, the mortality rate of the identified coinfection was 98 (25.39%), similar to the 26% case fatality rate reported by the World Health Organization (WHO) and the Pan American Health Organization (PAHO) in 2018 [9], and exceeding the national prevalence of 10.8% described in Colombia by other authors [10]. Nevertheless, in different areas of the country, such as the central region, prevalence reached 38.9% [6]. This suggests that mortality among patients coinfected with TB/HIV has increased over time, which correlates with higher rates of HIV and TB diagnoses in Colombia and worldwide. On the other hand, this increase in mortality can also be explained by the greater immunological deterioration of patients due to abandonment of antiretroviral and antitubercular treatment, aging leading to higher comorbidity in the general population, increased psychoactive substance use, and certain social barriers to accessing healthcare.

In addition, the present study demonstrates that 44 (11.4%) cases had previously been treated for TB, showing a high rate of non-adherence to antiretroviral and anti-TB therapy, which leads to increased drug resistance and higher mortality. The rates and causes of treatment dropout require additional examination in future studies to inform the deployment of therapies targeting this issue.

Regarding social determinants, the following were identified: 49 (12.69%) of patients showed signs of malnutrition, 14 (3.63%) were not affiliated with any health insurance plan, and 43 (11.14%) belonged to groups with unfavorable socioeconomic conditions (14 migrants, 3.63%; 21 prisoners, 5.44%; eight indigenous, 2.07%). These groups have unique risk factors that increase their vulnerability to TB/HIV coinfection and provide situations that hasten lethal results. This conclusion is supported by the findings of a study conducted in the Valle Department (Colombia) [11], which found that not belonging to these more vulnerable population groups improves survival from TB/HIV coinfection by providing better healthcare and access to health services. It is important to stress that, while poor nutritional condition is a risk factor for TB/HIV coinfection, it is frequently the result of severe disease.

Some studies in Colombia [12] and other South American countries [13] describe that, due to their immune deterioration, patients living with HIV are at greater risk of developing extrapulmonary TB conditions. However, this study found that HIV-positive patients had a higher incidence of pulmonary TB (300; 77.72%), compared to 86 (22.28%) patients who developed extrapulmonary TB. About extrapulmonary TB conditions, this study found that the most frequent were lymph node (39; 10.10%), pleural (19; 4.92%), and meningeal (16; 4.15%) forms, which is consistent with the findings described by previous research in Colombia [14] and other countries [15]. This finding is relevant, as meningeal TB has been described as the most lethal form of TB, with high mortality in hospitalized patients, especially when there are delays in the starting treatment [15,16].

In terms of diagnosis, clinical criteria were used in 204 (52.85%) of the cases, which is consistent with the Colombian Ministry of Health's diagnostic algorithm [2]. The clinical approach is the first step in the diagnosis since it is less expensive and more accessible, particularly in places where advanced diagnostic technology is lacking. Smear microscopy was the most employed laboratory approach (256; 67.09%) to confirm the diagnosis of TB in cases of clinical suspicion, followed by culture (125; 32.39%) and finally molecular testing (85; 22.02%).

Smear microscopy was found to be the diagnostic test with the highest number of positive results for TB in patients living with HIV, which contrasts with other studies, where more negative results have been obtained in smear microscopy in patients coinfected with TB/HIV compared to patients without coinfection [17,18]. The lack of access to other diagnostic methods or more expensive technologies in many regions of Colombia may explain why smear microscopy is the most used technique for TB diagnosis.

In the records analyzed, *Mycobacterium tuberculosis* and *Mycobacterium bovis* were identified in 60 (15.54%) and four (1.04%) of the confirmed TB cases, respectively. In the remaining 322 (83.42%), the specific mycobacterial species was not identified, as the diagnosis relied on clinical criteria or smear microscopy. This finding contrasts with other studies in Colombia, which have reported Mycobacterium avium and non-tuberculous mycobacteria as the most common etiological agents of TB in patients living with HIV [19,20]. It is important to note that this research found that very few molecular and microbiological diagnostic tests, including smear microscopy, were conducted. Such tests are beneficial for the timely diagnosis of HIV-positive patients in regions with a high prevalence of TB, as recommended by other studies in Colombia [19] and other countries [21]. This situation suggests that the TB management program in the department of Cordoba should enhance the use of these testing methods in diagnosing TB.

In this study, the bivariate analysis performed using the chi-square test found that the variables “hospitalization” and “type of case confirmation” are statistically significantly associated (p < 0.05) with the event of interest. This suggests that these two conditions are associated with a higher risk of mortality in patients with TB/HIV coinfection. These findings coincide with reports from studies conducted in other countries, which describe TB as one of the leading causes of mortality in HIV-positive patients who are hospitalized [22,23] and that microbiological confirmation of TB is associated with a higher risk of mortality in patients living with HIV [24]. This indicates that patients with greater immune compromise due to HIV have more advanced forms of TB that require hospitalization and develop other coinfections (histoplasmosis, pneumocystis, toxoplasmosis, and cytomegalovirus, among others) that increase the likelihood of death. However, due to the limitations of our study, it was not possible to evaluate other coinfections that may be a cause of death in HIV-positive patients diagnosed with non-severe TB.

The reduced explanatory model selected in the multivariate analysis contains the variables “hospitalization,” “histopathology result,” “smear microscopy result,” and “type of case confirmation.” When analyzing each of the coefficients in this model, it can be inferred that when the variables “histopathology result,” “smear microscopy result,” and “type of case confirmation” are held constant, hospitalization makes it 17.89 times more likely that a patient with TB/HIV coinfection will die. When the variables “hospitalization,” “histopathology result,” and “case confirmation type” are fixed, a positive smear test result makes it 0.6 times more likely that a patient with TB/HIV coinfection will die. When the variables “hospitalization,” “smear microscopy result,” and “type of case confirmation” are fixed, a positive histopathology result makes it 17.86 times more likely that a patient with TB/HIV coinfection will die. When the variables “hospitalization,” “histopathology results,” and “smear microscopy result” are fixed, confirmation of TB diagnosis by microbiological tests makes it 0.66 times less likely that an HIV-positive patient will die. This suggests that TB diagnosis based solely on clinical criteria leads to misleading results, reflecting an increase in false negative rates, such that many patients avoid treatment, leading to an increase in mortality. In addition, it is suggested that urine lipoarabinomannan lateral flow tests (LF-LAM) be performed, as their speed and simplicity facilitate the diagnosis of active TB in resource-limited settings, where sputum testing may be difficult to accomplish.

When analyzing the RP values for each variable in the selected explanatory model, in the variables “type of case confirmation” (p-value 0.033) and “smear microscopy result” (p-value 0.041), a statistically significant association with mortality was found . This indicates that in HIV-positive patients, the risk of death is 0.51 (CI 0.27-0.84) times lower when TB is diagnosed using microbiological criteria compared to those in whom the diagnosis is made solely on clinical criteria, probably because the patient may have another infection that has not been identified and treated. It also suggests that in HIV-positive patients, the risk of death is 1.82 (CI 1.02-3.26) times higher in those who test positive in smear microscopy than in those who test negative in this laboratory test. These findings are consistent with reports from studies conducted on the African continent [22].

The selected explanatory model demonstrates good accuracy, indicating that it effectively identifies a substantial proportion of patients who ultimately die. While it demonstrates low sensitivity, its high specificity indicates its ability to accurately identify TB/HIV coinfected patients at low risk of mortality. In other words, negative results from the variables outlined in the model suggest a high likelihood of survival. The ROC curve illustrates that the model possesses strong explanatory power regarding mortality, with an AUC of 76.4% (95% CI 71.7-81.0%), indicating that it significantly outperforms a random model. The implications of these findings are expected to be especially beneficial for health institutions in the department of Córdoba, where high prevalence and mortality rates from TB/HIV coinfection have been observed compared to other regions of the country.

The limitations of this study include the following: First, there are issues related to the retrospective nature of the data collected. Inadequate completion of epidemiological notification forms and the absence of additional information hinder the assessment of patients' immunovirological status (including viral loads and CD4 cell counts), the antiretroviral treatments administered, resistance to antifungal therapy, coinfections, or other concurrent diagnoses, as well as other treatments received. This information is crucial, as it significantly impacts mortality in cases of TB/HIV coinfection. Second, the study lacks a temporal follow-up of patients. The absence of longitudinal data prevents a comprehensive understanding of the influence of transient or intermittent risk factors associated with mortality, such as complications from concurrent infections, relapses due to decompensated chronic conditions, and exacerbations of TB/HIV coinfection resulting from treatment abandonment, among other factors. Third, despite improvements in surveillance and data collection by the TB control program in Córdoba, there has been a history of underreporting for several years. This suggests that the prevalence of coinfection could be higher than reported, which also limits the external validity of the findings, as they do not accurately reflect the general population's situation in the department.

## Conclusions

This is the first study in the Department of Córdoba to describe the behavior of TB/HIV coinfection and evaluate sociodemographic characteristics, clinical aspects, and diagnostic findings as risk factors associated with mortality. This area of Colombia has a high prevalence of TB/HIV coinfection, above the national average; it also has a high mortality rate, being one of the areas of the country with the highest numbers. The population groups at highest risk of mortality are males, young adults, and people in disadvantaged socioeconomic situations. Hospitalization and clinical confirmation of TB are risk factors that increase mortality. The low number of microbiological diagnoses performed indicates underreporting of TB in this area of the country. Further studies using more advanced microbiological and histopathological techniques are recommended to improve the diagnosis of TB/HIV coinfection and reduce mortality rates. Additional research is needed, implementing different design techniques and fewer limitations, to corroborate the findings of this study.
